# A MYB transcription factor, DcMYB6, is involved in regulating anthocyanin biosynthesis in purple carrot taproots

**DOI:** 10.1038/srep45324

**Published:** 2017-03-27

**Authors:** Zhi-Sheng Xu, Kai Feng, Feng Que, Feng Wang, Ai-Sheng Xiong

**Affiliations:** 1 State Key Laboratory of Crop Genetics and Germplasm Enhancement, College of Horticulture, Nanjing Agricultural University, Nanjing, 210095, China

## Abstract

Carrots are widely grown and enjoyed around the world. Purple carrots accumulate rich anthocyanins in the taproots, while orange, yellow, and red carrots accumulate rich carotenoids in the taproots. Our previous studies indicated that variation in the activity of regulatory genes may be responsible for variations in anthocyanin production among various carrot cultivars. In this study, an R2R3-type MYB gene, designated as *DcMYB6*, was isolated from a purple carrot cultivar. In a phylogenetic analysis, DcMYB6 was grouped into an anthocyanin biosynthesis-related MYB clade. Sequence analyses revealed that DcMYB6 contained the conserved bHLH-interaction motif and two atypical motifs of anthocyanin regulators. The expression pattern of *DcMYB6* was correlated with anthocyanin production. *DcMYB6* transcripts were detected at high levels in three purple carrot cultivars but at much lower levels in six non-purple carrot cultivars. Overexpression of *DcMYB6* in *Arabidopsis* led to enhanced anthocyanin accumulation in both vegetative and reproductive tissues and upregulated transcript levels of all seven tested anthocyanin-related structural genes. Together, these results show that DcMYB6 is involved in regulating anthocyanin biosynthesis in purple carrots. Our results provide new insights into the regulation of anthocyanin synthesis in purple carrot cultivars.

Carrots (*Daucus carota* L.; 2n = 2x = 18) are an economically important root crop worldwide. The taproot of cultivated carrots exhibits a range of colors including orange, yellow, red, white, and purple. Purple carrots contain anthocyanins, whereas the orange, red, and yellow pigmentation of carrot taproots is due to carotenoids^1^. White carrots contain very low levels of carotenoids^2^. Thus, cultivated carrots can be divided into two distinct groups: the anthocyanin or eastern group (*Daucus carota* ssp. *sativus* var. *atrorubens* Alef.) and the carotene or western group (*Daucus carota* ssp. *sativus* var. *sativus*)^3^.

Anthocyanins are a group of flavonoids that perform various important functions in plants. They provide pigmentation in vegetative and reproductive tissues^4^,^5^,^6^,^7^,^8^, enhance cold, drought, and salt tolerance^9^,^10^,^11^, and protect against damage from ultraviolet light, insect herbivory, and pathogen attack^12^,^13^. Purple carrots accumulate rich anthocyanins in the fleshy taproot. The role of anthocyanins in carrot taproots is unclear; however, it is conceivable that they could protect the taproot from insect and pathogen attack. Anthocyanins are beneficial to human health^14^ and are used as natural food colorants in beverages, candies, and ice cream^15^.

The genetics of anthocyanin biosynthesis have been extensively studied in many plant species. Regulatory genes encoding transcription factors (TFs) control the transcription of structural genes encoding enzymes involved in anthocyanin biosynthesis. In our previous studies, the transcripts of numerous structural genes in the anthocyanin pathway were undetectable or barely detectable in the taproots of non-purple carrots, but were detected at high levels in those of purple carrots. It seems likely that variation in the activity of regulatory genes is the key factor determining anthocyanin production in carrots^16^.

In many species studied to date, the R2R3-MYB TFs, basic helix-loop-helix (bHLH) TFs, and WD-repeat (WDR) proteins form ‘MBW’ complexes that bind to the promoters of target genes to directly activate the transcription of structural genes in the anthocyanin pathway^17^. The MYB proteins in this complex are often the key component determining variation in anthocyanin production^18^,^19^,^20^. Anthocyanin-related MYBs have been identified in many plant species, for example, *Arabidopsis* AtMYB75 (PAP1), AtMYB90 (PAP2), AtMYB113, and AtMYB114^21^, *Vitis vinifera* VvMYB1a^22^, *Ipomoea batatas* IbMYB1^8^, and *Malus × domestica* MdMYB10, MdMYB1/MdMYBA^19^,^23^,^24^. Overexpression of genes encoding these MYB TFs in heterologous or homologous plant species leads to enhanced anthocyanin accumulation.

In this study, a gene encoding an R2R3-type MYB, designated as *DcMYB6*, was isolated from a purple carrot cultivar. The correlation between its expression with anthocyanin production in purple and non-purple carrots was analyzed. The function of *DcMYB6* was also analyzed by overexpression in *Arabidopsis* plants. These results will further our understanding of how anthocyanin synthesis is regulated in carrots.

## Results

### Sequence analysis of *DcMYB6*

The amplification products of the genomic DNA sequence and the open reading frame (ORF) sequence of *DcMYB6* from the carrot cultivar ‘Deep purple’ are shown in Supplementary Fig. S1. The genomic DNA sequence of *DcMYB6* was 1,801 bp long while the ORF sequence of *DcMYB6* was 903 bp long, encoding a polypeptide of 300 amino acids (Supplementary Fig. S1A). Alignment analysis of genomic DNA and ORF sequences revealed that the *DcMYB6* gene consisted of two introns and three exons (Supplementary Fig. S1B).

**Figure 1:**
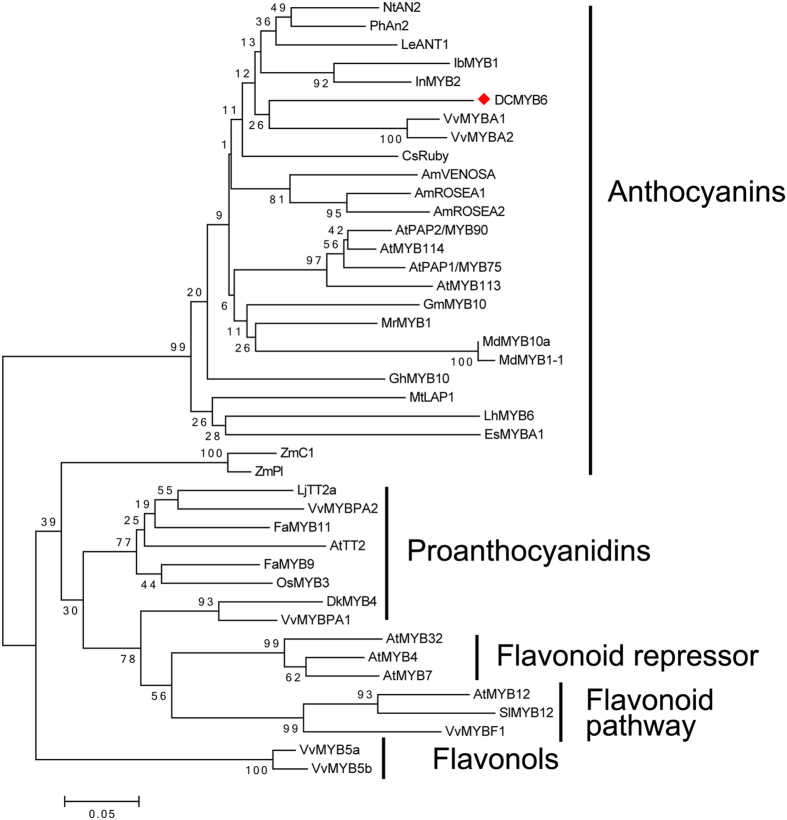
Phylogenetic relationships among DcMYB6 and flavonoid-related R2R3-MYBs from other plant species. Phylogenetic tree was built using the neighbor-joining method using MEGA 5 software; bootstrap value was set to 1000. Red diamond indicates DcMYB6. Putative functions of all R2R3-MYBs are listed on the right.

We conducted a phylogenetic analysis with the deduced amino acid sequences of *DcMYB6* and other R2R3-MYB TFs involved in the biosynthesis of different secondary metabolites. Supplementary Table S1 lists the GenBank accession numbers of the R2R3-MYBs used to build the phylogenetic tree. In the phylogenetic tree, R2R3-MYB TFs with similar functions clustered together, and DcMYB6 grouped into an anthocyanin biosynthesis-related MYB clade (Fig. 1), which included tobacco (*Nicotiana tabacum*) NtAN2, petunia (*Petunia hybrida*) PhAn2, tomato (*Lycopersicon esculentum*) LeANT1, sweet potato (*I. batatas*) IbMYB1, morning glory (*I. nil*) InMYB2, grapevine (*V. vinifera*) VvMYBA1 and VvMYBA2, blood orange (*Citrus sinensis*) CsRuby, snapdragon (*Antirrhinum majus*) AmVENOSA, AmROSEA1, and AmROSEA2, *Arabidopsis thaliana* AtPAP1, AtPAP2, and AtMYB114, mangosteen (*Garcinia mangostana*) GmMYB10, Chinese bayberry (*Myrica rubra*) MrMYB1, apple (*Malus × domestica*) MdMYB10a and MdMYB1-1, *Gerbera hybrida* GhMYB10, *Medicago truncatula* MtLAP1, *Lilium hybrid* LhMYB6, and *Epimedium sagittatum* EsMYBA1.

**Figure 2:**
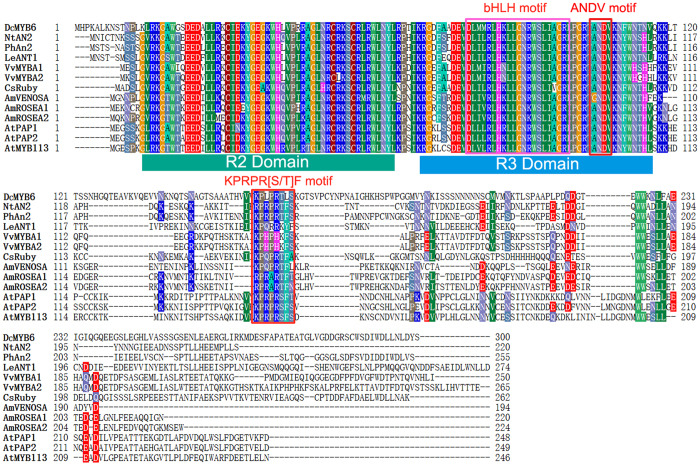
Alignment of deduced amino acid sequence of DcMYB6 and R2R3-MYB proteins from other plant species. Alignment was conducted using BioEdit (Version 7.0.1). Identical amino acid residues are shaded as per color table (threshold for shading was set to 60%). R2 and R3 domains are indicated. Boxes show BHLH, ANDV and KPRPR[S/T]F motifs.

Next, we conducted an alignment analysis of the deduced amino acid sequence of DcMYB6 with those of other MYB TFs related to anthocyanin biosynthesis. Like other MYB TFs, DcMYB6 contained the highly conserved R2R3 domain at the N-terminus (Fig. 2). DcMYB6 showed high sequence homology with other MYB TFs within the R2R3 domain, sharing the highest identity (85%) with LeANT1 and the lowest identity (80%) with AmVENOSA. However, all the MYB TFs showed little homology in the C-terminus sequence to the R2R3 domain. When whole sequences were compared, DcMYB6 shared the highest identity (44%) with PhAn2 and the lowest identity (32%) with AmROSEA1.

The alignment showed that the [D/E]Lx2[R/K]x3Lx6Lx3 R motif, also known as the bHLH motif^25^, which is required for the interaction with bHLH proteins, was present in the R3 domain of all the analyzed MYB TFs (Fig. 2). The conserved ANDV motif that has been identified in MYB TFs in the anthocyanin pathway in the Rosaceae^26^ was also present in all of the analyzed MYB TFs and was modified to [A/G]NDV. Besides these motifs, the MYB TFs contained the motif KPRPR[S/T]F defined by Stracke *et al*.^27^, which was modified to [K/R]Pxx[H/R] [K/S/T][F//L/Y], in the C-terminal region.

### Quantitative real-time PCR analysis of *DcMYB6* in purple and non-purple carrot taproots

**Figure 3:**
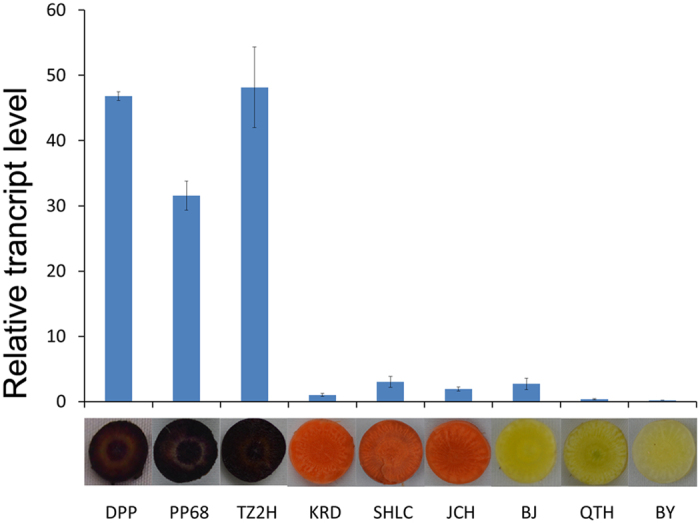
Transcript profiles of *DcMYB6* in 90-day-old taproots of three purple and six non-purple carrot cultivars. Data represent means of three biological replicates ± SD. Cultivar abbreviations: DPP, Deep purple; PP68, Purple 68; TZ2H, Tianzi2hao; KRD, Kuroda; SHLC, Sanhongliucun; JCH, Junchuanhong; BJ, Bejo1719; QTH, Qitouhuang; BY, Baiyu.

At the 90-day-old stage, purple carrot cultivars had accumulated rich anthocyanins whereas anthocyanins were barely detectable in, or absent from non-purple carrot cultivars^16^. Using specific primer pairs, qRT-PCR analyses were performed to quantify the transcript levels of *DcMYB6* in purple and non-purple carrots at this stage. The transcript levels of *DcMYB6* in the taproots of three purple carrot cultivars (‘Deep purple’, ‘Purple 68’, and ‘Tianzi2hao’) were approximately 10–229-fold higher than those in the taproots of six non-purple carrot cultivars (‘Kuroda’, ‘Sanhongliucun’, ‘Junchuanhong’, ‘Bejo1719’, ‘Qitouhuang’, and ‘Baiyu’). Among the three purple carrot cultivars, ‘Tianzi2hao’ had the highest transcript level of *DcMYB6* and ‘purple 68’ had the lowest. Among the six non-purple carrot cultivars, ‘Baiyu’ had the lowest transcript level of *DcMYB6* and ‘Sanhongliucun’ had the highest (Fig. 3).

### Subcellular localization of DcMYB6 protein

**Figure 4:**
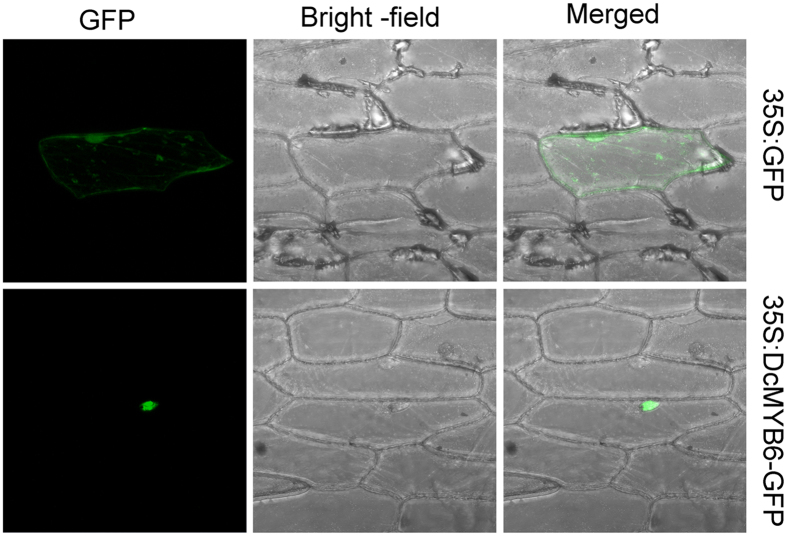
Subcellular localization of GFP fusions of DcMYB6. Onion epidermal cells transiently expressing GFP and DcMYB6-GFP under the control of the CaMV 35 S promoter.

To investigate the subcellular localization of DcMYB6, the *DcMYB6* coding sequence was fused in-frame to the 5′ terminus of the gene encoding GFP, and the construct was transiently expressed in onion cells. In onion cells expressing GFP alone, fluorescence was localized in the cytoplasm and nucleus (Fig. 4 up). Onion cells expressing the DcMYB6-GFP fusion protein showed a strong signal in the nucleus (Fig. 4 down).

### Overexpression of *DcMYB6* in transgenic *Arabidopsis* induced anthocyanin production

**Figure 5:**
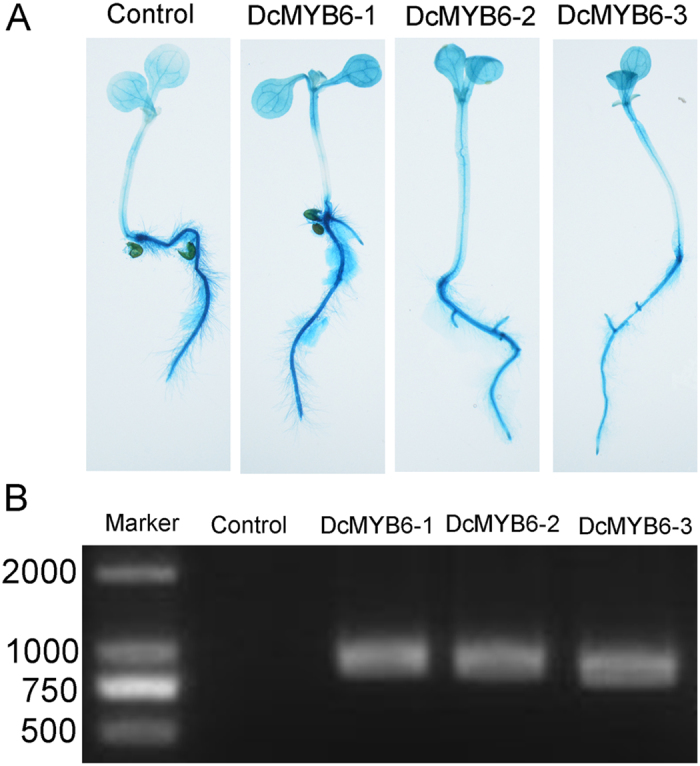
Identification of transgenic *Arabidopsis* plants with histochemical GUS activity and PCR analyses. (**A**) Histochemical GUS activity analysis of transgenic *Arabidopsis* plants overexpressing empty vector (control) and *DcMYB6* (DcMYB6-1, DcMYB6-2, and DcMYB6-3). (**B**) PCR-amplified *DcMYB6* fragments from the same transgenic *Arabidopsis* plants as above.

The *DcMYB6* gene driven by the CaMV 35 S promoter was overexpressed in *Arabidopsis* plants to test its function. *Arabidopsis* seedlings of three homozygous CaMV 35 S:DcMYB6 transgenic lines (DcMYB6-1, DcMYB6-2, and DcMYB6-3) and one control transgenic line, which were selected on MS agar plates containing hygromycin, showed β-glucuronidase (GUS) activity (Fig. 5A). A PCR product of approximately 900 bp corresponding to the DcMYB6 coding sequence was detected in all three CaMV 35 S:DcMYB6 transgenic *Arabidopsis* lines analyzed, whereas no such PCR product was amplified from control transgenic plants (Fig. 5B).

**Figure 6:**
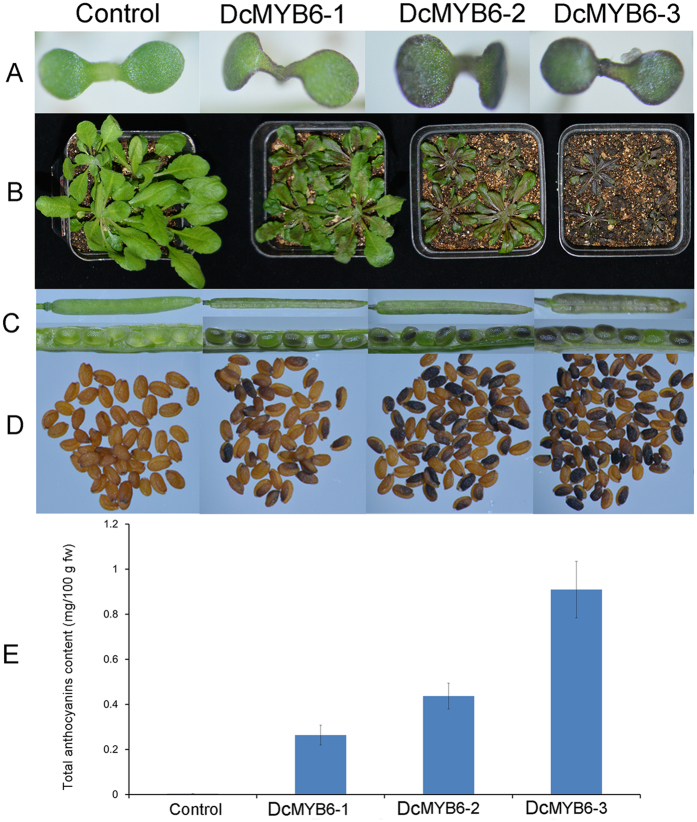
Functional analysis of DcMYB6 in *Arabidopsis*. (**A** and **B**) Ten-day-old and 40-day-old transgenic *Arabidopsis* plants overexpressing empty vector (control) and *DcMYB6* (DcMYB6-1, DcMYB6-2, and DcMYB6-3). (**C**) Immature capsules and seeds from control and three lines of transgenic *Arabidopsis* plants overexpressing DcMYB6. (**D**) Mature seeds from control and three lines of *DcMYB6*-overexpressing transgenic *Arabidopsis* plants. (**E**) Total anthocyanin contents of 40-day-old transgenic *Arabidopsis* plants overexpressing empty vector (control) and *DcMYB6* (DcMYB6-1, DcMYB6-2, and DcMYB6-3).

Overexpression of *DcMYB6* in *Arabidopsis* induced anthocyanin accumulation. Compared with control plants, DcMYB6-1, DcMYB6-2, and DcMYB6-3 plants exhibited dark-purple pigments in the leaves, siliques, and immature and mature seed coats (Fig. 6A–D), and delayed growth. The total anthocyanin content in the whole plants of DcMYB6-1, DcMYB6-2, and DcMYB6-3 plants was approximately 66–228-fold higher than that incontrol plants (Fig. 6E). Among the three CaMV 35 S:DcMYB6 transgenic *Arabidopsis* lines, DcMYB6-1 showed the lowest total anthocyanin content and DcMYB6-3 showed the highest.

### Up-regulation of anthocyanin biosynthetic genes in transgenic *Arabidopsis* overexpressing *DcMYB6*

**Figure 7:**
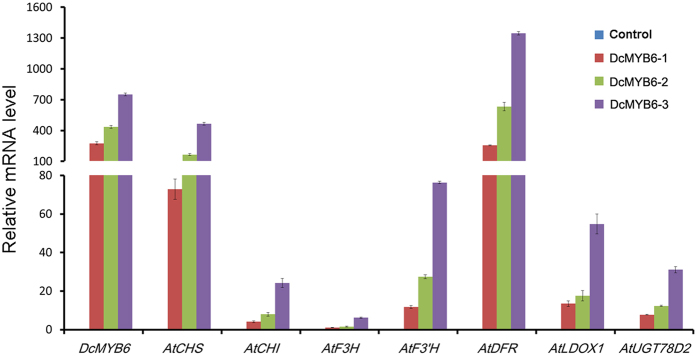
Transcript levels of *DcMYB6* and seven anthocyanin pathway structural genes in transgenic *Arabidopsis* plants. Gene transcript levels in *Arabidopsis* plants overexpressing empty vector (control) and *DcMYB6* (DcMYB6-1, DcMYB6-2, and DcMYB6-3) were detected by quantitative RT-PCR. Data represent means of three biological replicates ± SD.

Among the three CaMV 35 S:DcMYB6 transgenic *Arabidopsis* lines, DcMYB6-1 plants showed the lowest *DcMYB6* transcript levels and DcMYB6-3 plants showed the highest (Fig. 7). As expected, *DcMYB6* transcripts were undetectable in the control *Arabidopsis* plants. The results of the qRT-PCR analyses also determined which endogenous anthocyanin pathway structural genes were up-regulated in the transgenic *Arabidopsis* plants overexpressing DcMYB6. Compared with the control line, the transgenic *Arabidopsis* plants overexpressing DcMYB6 showed significantly increased transcript levels of *AtCHS* (chalcone synthase), *AtCHI* (chalcone isomerase), *AtF3H* (flavanone 3- hydroxylase), *AtF3′H* (flavonoid 3′-hydroxylase), *AtDFR* (dihydroflavonol 4- reductase), *AtLDOX* (leucoanthocyanidin dioxygenase), and *AtUGT78D2* (Fig. 7). Among the three transgenic *Arabidopsis* lines overexpressing *DcMYB6*, DcMYB6-1 plants showed the lowest transcript levels of these structural genes and DcMYB6-3 plants showed the highest. Transcripts of these structural genes were undetectable or barely detectable in the control *Arabidopsis* plants.

## Discussion

Anthocyanins are water-soluble pigments responsible for purple colors in carrots. In plants, TFs such as MYB, bHLH, and WD40 upregulate the expression of structural genes in the anthocyanin biosynthesis pathway. Two previous studies showed that the expression levels of all anthocyanin pathway structural genes were significantly lower in non-purple carrot cultivars than in purple carrot cultivars, which possibly resulted from the inactivation of regulator genes^16^,^28^. In other plant species, many R2R3-MYB TFs are known to control anthocyanin biosynthesis by regulating structural genes in the anthocyanin pathway^4^,^16^,^17^,^22^,^25^. However, little is known about the R2R3-MYB TFs involved in regulating the anthocyanin pathway in carrot. A previous study reported that DcMYB3 and DcMYB5 might upregulate the activity of the *DcPAL3* promoter^29^. In the present study, a gene encoding R2R3-MYB, namely *DcMYB6*, was isolated from ‘Deep purple’, a purple carrot cultivar.

DcMYB6 grouped into the same clade as the MYB TF family of the anthocyanin pathway, and shared high identity with anthocyanin-regulating MYB TFs from other species within the R2R3 domain. DcMYB6 was found to contain the conserved bHLH interaction motif [D/E]Lx2[R/K]x3Lx6Lx3 R in the R3 domain, and an atypical anthocyanin regulator motif KPRPR[S/T]F at the C-terminus. Another conserved motif, [A/G]NDV, which distinguishes anthocyanin and non-anthocyanin MYB TFs in the Rosaceae, was also found in DcMYB6. The presence of these motifs suggested that DcMYB6 may be involved in regulating anthocyanin biosynthesis. In several other plant species, the expression of many *R2R3-MYB* genes in the anthocyanin pathway is strong correlated with anthocyanin accumulation. For example, *MdMYB10* was found to be highly expressed highly in red-fleshed apple and in the colored skin of white-fleshed apple, but was virtually undetectable in the white cortex of white-fleshed apple^19^. In the present study, *DcMYB6* transcript levels corresponded well with anthocyanin pigmentation; there were much higher transcript levels in all three 90-day-old purple carrot taproots than in 90-day-old taproots of the six non-purple carrot cultivars. Therefore, DcMYB6 is probably involved in regulating anthocyanin biosynthesis in purple carrot taproots.

The reason why *DcMYB6* transcript levels were much lower in non-purple carrots than in purple carrots is still unknown. In peach (*Prunus persica*), the heterodimer of BL and PpNAC1 was shown to activate transcription of the anthocyanin-related MYB, *PpMYB10.1*^30^. In European pear (*Pyrus communis*), methylation of the PcMYB10 promoter reduced *PcMYB10* expression levels and resulted in a peel color change from red to green^31^. Insertions and deletions in the promoter region have been shown to affect the expression levels of anthocyanin-related MYBs in some species, such as apple (*Malus* × *domestica*) and grapevine (*V. vinifera*)^20^,^22^. In future work, we will attempt to establish the reason for the different transcript level of *DcMYB6* in purple and non-purple carrots.

In several studies, overexpression of anthocyanin-related MYB TFs in heterologous plant species led to enhanced anthocyanin accumulation^32^,^33^. In this study, transgenic *Arabidopsis* plants overexpressing *DcMYB6* exhibited a clearly darker color and accumulated higher levels of anthocyanins in both vegetative and reproductive tissues, compared with those in control *Arabidopsis* plants. Furthermore, qRT-PCR analyses of the three transgenic *Arabidopsis* lines with different transcript levels of *DcMYB6* and different total anthocyanin levels showed that higher transcript levels of *DcMYB6* led to greater anthocyanin accumulation. Also, the transcript levels of all seven tested anthocyanin-related structural genes were much higher in transgenic *Arabidopsis* plants overexpressing *DcMYB6* than in control *Arabidopsis* plants. Together, these results indicate that DcMYB6 could enhance anthocyanin accumulation in *Arabidopsis* by upregulating anthocyanin-related structural genes, and suggest that DcMYB6 regulates anthocyanin biosynthesis in purple carrots.

In conclusion, an R2R3-MYB TF, DcMYB6, was isolated from a purple carrot cultivar and was found to be involved in regulating the anthocyanin biosynthetic pathway. The results of this study provide important information on the pigmentation of purple carrots. Other TFs such as bHLH and WD40 that form complexes with MYB proteins and together regulate anthocyanin biosynthesis have not yet been identified in carrots. In future work, we will test whether overexpression of *DcMYB6* in non-purple carrot cultivars leads to anthocyanin accumulation.

## Methods

### Plant materials

Three purple carrot cultivars (‘Deep purple’, ‘Purple 68’, and ‘Tianzi2hao’), three orange carrot cultivars (‘Kuroda’, ‘Sanhongliucun’, and ‘Junchuanhong’), and three yellow carrot cultivars (‘Bejo1719’, ‘Qitouhuang’, and ‘Baiyu’), which are widely cultivated in China, were chosen for this work. Seeds were grown in a controlled artificial climatic chamber under the same conditions as previously described^16^. *Arabidopsis thaliana* ecotype Columbia was grown under the same conditions.

### RNA and DNA extraction from carrots and cDNA preparation

Total RNA was extracted from taproots of 90-day-old carrot plants using an RNAsimple Total RNA Kit (Tiagen, Beijing, China). First-strand cDNA was synthesized using the PrimeScript™ RT reagent kit with gDNA Eraser (Perfect Real Time; Takara, Dalian, China). cDNA was diluted 20-fold for gene cloning and qRT-PCR analyses. Genomic DNA was isolated from young leaves with a DNAsecure plant kit (Tiangen).

### Isolation of genomic DNA and cDNA sequence of *DcMYB6*

AtPAP1 (AAG42001) was BLASTed against our CarrotDB: a genomic and transcriptomic database for carrot^34^ and the high-quality carrot genome which spans 421.5 Mb and accounts for ~90% of the estimated genome size (473 Mb)^35^. Two transcript contigs showing high sequence identity with AtPAP1 and with higher FPKM values in purple carrots than in non-purple carrots were identified in the transcriptomic database of CarrotDB. After assembling these two transcript contigs, an ORF of 903 bp was identified and predicted to be a MYB TF using Pfam (http://pfam.xfam.org/). This MYB TF was designated as DcMYB6 in this study. Two genomic sequence scaffolds (scaffold 016995 and scaffold 029424) that matched the ORF sequence were identified in the genomic database of CarrotDB. However, no genomic sequence matching the ORF sequence was found in the high-quality carrot genome.

The ORF sequence of DcMYB6 was amplified from cDNA produced from 90-day-old ‘Deep purple’ carrot taproots using PrimeSTAR HS DNA polymerase (Takara, Otsu, Japan) with the forward primer (5′-CGCGCGGATCTTCCAGAGATTATGCATCCAAAGGCTTTGAAGAAT-3′) and reverse primer (5′-CACGCCTGCCGTTCGACGATTTTAACTATAATCCAAGTTAAGAAGGTCCC-3′). The ORF sequence was then cloned into the pMD19-T simple vector (Takara, Otsu, Japan) using the ClonExpress II One Step Cloning Kit (Vazyme Biotech Co. Ltd., Nanjing, China) before sequencing (Genscript, Nanjing, China). The same pairs of primers were also used to amplify the genomic clone of *DcMYB6* from genomic DNA extracted from carrot leaves. The full-length ORF and DNA sequences of *DcMYB6* have been deposited in the GenBank database under the accession numbers KY020445 and KY020446, respectively.

### Subcellular localization analysis

The protein-coding region of *DcMYB6* was amplified with the forward primer (5′- CACCATCACCATCACGCCATGATGATCAAGAGCACTGGTAATCC-3′) and the reverse primer (5′- CACTAGTACGTCGACCATGGCACTATAGTCCTGGTTGAGAAGATCCC-3′), and was subcloned into the pA7-GFP vector at the *Nco* I site to create the CaMV 35 S:DcMYB6-GFP fusion construct. This construct and the pA7-GFP empty vector (as control) were both bombarded into onion epidermal cells using a Biolistic PDS-1000 instrument (Bio-Rad, Hercules, CA, USA). After incubation at 25 °C for at least 16 h in the dark, samples were observed under a confocal laser scanning microscope.

### Overexpression vector construct preparation and *Arabidopsis* transformation

The coding sequence of *DcMYB6* was amplified with the forward primer (5′-TTTACAATTACCATGGGATCCATGCATCCAAAGGCTTTGAAGAAT-3′) and the reverse primer(5′-ACCGATGATACGAACGAGCTCTTAACTATAATCCAAGTTAAGAAGGTCCC-3′), and then subcloned into the binary vector pCAMBIA-1301 under the control of the CaMV 35 S promoter and the pea rbcSE9 terminator to create the CaMV 35 S:DcMYB6 construct. This construct was introduced into *Agrobacterium tumefaciens* strain GV3101 by electroporation and then transformed into *Arabidopsis* using the floral-dip method^36^. Transgenic *Arabidopsis* plants carrying the *DcMYB6* gene were identified by selection on half-strength Murashige and Skoog (MS) agar plates containing 35 mg/L hygromycin, assaying for GUS activity, and detecting the presence of the transgene by reverse transcription PCR with the forward primer (5′-ATGCATCCAAAGGCTTTGAAGAAT-3′) and the reverse primer (5′-AAGCACAACAAATGGTACAAG-3′), which were designed according to the sequence of DcMYB6 and the pea rbcSE9 terminator, respectively. Three transgenic *Arabidopsis* lines (DcMYB6-1, DcMYB6-2, and DcMYB6-3) with black leaves were used for further experiments. *Arabidopsis* plants transformed with the pCAMBIA-1301 empty vector served as controls.

### Determination of total anthocyanin content

Total anthocyanins were extracted from 40-day-old transgenic *Arabidopsis* plants (T3) as described previously^37^. Total anthocyanin quantities are presented in mg cyanidin 3-*O*-glycoside equivalents per 100 g fw (mg/100 g fw). Three biological replicates were analyzed for each sample.

### Quantitative real-time PCR expression analysis

The mRNA levels of the *DcMYB6* gene in 90-day-old carrot taproots and in 40-day-old transgenic *Arabidopsis* plants were determined by qRT-PCR with the forward primer (5′-GCCATAGGGCACAAGCACTCT-3′) and the reverse primer (5′-GATCCCAATTTCCGCAAACAA-3′). Total RNA was extracted from 40-day-old transgenic *Arabidopsis* plants and used to synthesize cDNA using the method described above. To determine the transcript levels of anthocyanin pathway structural genes in transgenic *Arabidopsis*, qRT-PCR assays were performed with the primers listed in Supplementary Table S2. The *DcActin1* gene was used as an internal standard in carrot with the same primers as described previously^16^,^38^, while the *AtActin2* gene was used as an internal standard for normalization in *Arabidopsis* and was amplified using the primers listed in Supplementary Table S2. Experiments were conducted using three biological replicates for each sample. The relative gene transcript level was calculated with the 2^−ΔΔCT^ method^39^. To compare *DcMYB6* expression patterns among purple and non-purple carrots at the 90-day-old stage, the ΔΔC_T_ was calculated by subtracting ΔC_T_ of ‘Kuroda’ from ΔC_T_ of all carrot cultivars. To compare the transcript levels of *DcMYB6* and anthocyanin pathway structural genes among transgenic *Arabidopsis* plants, the ΔΔC_T_ was calculated by subtracting ΔC_T_ of the *AtF3H* (flavanone 3-hydroxylase) gene in *DcMYB6-1 Arabidopsis* plants from the ΔC_T_ of all tested genes in transgenic *Arabidopsis* plants.

## Additional Information

**How to cite this article:** Xu, Z.-S. *et al*. A MYB transcription factor, DcMYB6, is involved in regulating anthocyanin biosynthesis in purple carrot taproots. *Sci. Rep.* **7**, 45324; doi: 10.1038/srep45324 (2017).

**Publisher's note:** Springer Nature remains neutral with regard to jurisdictional claims in published maps and institutional affiliations.

## Supplementary Material

Supplementary Information

## References

[b1] Clotault J. . Expression of carotenoid biosynthesis genes during carrot root development. J Exp Bot 59, 3563–3573 (2008). 18757491 10.1093/jxb/ern210

[b2] Surles R. L., Weng N., Simon P. W. & Tanumihardjo S. A. Carotenoid profiles and consumer sensory evaluation of specialty carrots (*Daucus carota*, L.) of various colors. J Agric Food Chem 52, 3417–3421 (2004). 15161208 10.1021/jf035472m

[b3] Kammerer D., Carle R. & Schieber A. Quantification of anthocyanins in black carrot extracts (*Daucus carota* ssp *sativus* var. *atrorubens* Alef.) and evaluation of their color properties. Eur Food Res Technol 219, 479–486 (2004).

[b4] Li P. . Regulation of anthocyanin and proanthocyanidin biosynthesis by *Medicago truncatula* bHLH transcription factor MtTT8. New Phytol 210, 905–921 (2016). 26725247 10.1111/nph.13816

[b5] Singh R. . The oil palm *VIRESCENS* gene controls fruit colour and encodes a R2R3-MYB. Nat Commun 5, 4106 (2014). 24978855 10.1038/ncomms5106 PMC4078410

[b6] Chagne D. . An ancient duplication of apple MYB transcription factors is responsible for novel red fruit-flesh phenotypes. Plant Physiol 161, 225–239 (2013). 23096157 10.1104/pp.112.206771 PMC3532254

[b7] Vimolmangkang S., Han Y., Wei G. & Korban S. S. An apple MYB transcription factor, *MdMYB3*, is involved in regulation of anthocyanin biosynthesis and flower development. BMC Plant Biol 13, 176 (2013). 24199943 10.1186/1471-2229-13-176 PMC3833268

[b8] Mano H., Ogasawara F., Sato K., Higo H. & Minobe Y. Isolation of a regulatory gene of anthocyanin biosynthesis in tuberous roots of purple-fleshed sweet potato. Plant Physiol 143, 1252–1268 (2007). 17208956 10.1104/pp.106.094425 PMC1820918

[b9] Nakabayashi R. . Enhancement of oxidative and drought tolerance in *Arabidopsis* by overaccumulation of antioxidant flavonoids. Plant J 77, 367–379 (2014). 24274116 10.1111/tpj.12388 PMC4282528

[b10] Ahmed N. U., Park J. I., Jung H. J., Hur Y. & Nou I. S. Anthocyanin biosynthesis for cold and freezing stress tolerance and desirable color in *Brassica rapa*. Funct Integr Genomics 15, 383–394 (2015). 25504198 10.1007/s10142-014-0427-7

[b11] Oh J. E., Kim Y. H., Kim J. H., Kwon Y. R. & Lee H. Enhanced level of anthocyanin leads to increased salt tolerance in arabidopsis *PAP1-D* plants upon sucrose treatment. J Korean Soc Appl Biol Chem 54, 79–88 (2011).

[b12] Karageorgou P. & Manetas Y. The importance of being red when young: anthocyanins and the protection of young leaves of *Quercus coccifera* from insect herbivory and excess light. Tree Physiol 26, 613–621 (2006). 16452075 10.1093/treephys/26.5.613

[b13] Bassolino L. . Accumulation of anthocyanins in tomato skin extends shelf life. New Phytol 200, 650–655 (2013). 24102530 10.1111/nph.12524

[b14] Butelli E. . Enrichment of tomato fruit with health-promoting anthocyanins by expression of select transcription factors. Nat Biotechnol 26, 1301–1308 (2008). 18953354 10.1038/nbt.1506

[b15] Netzel M. . Cancer cell antiproliferation activity and metabolism of black carrot anthocyanins. Innov Food Sci Emerg 8, 365–372 (2007).

[b16] Xu Z. S. . Transcript profiling of structural genes involved in cyanidin-based anthocyanin biosynthesis between purple and non-purple carrot (*Daucus carota* L.) cultivars reveals distinct patterns. BMC Plant Biol 14 (2014). 10.1186/s12870-014-0262-y PMC4190390 25269413

[b17] Baudry A. . TT2, TT8, and TTG1 synergistically specify the expression of BANYULS and proanthocyanidin biosynthesis in *Arabidopsis thaliana*. Plant J 39, 366–380 (2004). 15255866 10.1111/j.1365-313X.2004.02138.x

[b18] Schwinn K. . A small family of MYB-regulatory genes controls floral pigmentation intensity and patterning in the genus *Antirrhinum*. Plant Cell 18, 831–851 (2006). 16531495 10.1105/tpc.105.039255 PMC1425845

[b19] Espley R. V. . Red colouration in apple fruit is due to the activity of the MYB transcription factor, MdMYB10. Plant J 49, 414–427 (2007). 17181777 10.1111/j.1365-313X.2006.02964.x PMC1865000

[b20] Espley R. V. . Multiple repeats of a promoter segment causes transcription factor autoregulation in red apples. Plant Cell 21, 168–183 (2009). 19151225 10.1105/tpc.108.059329 PMC2648084

[b21] Gonzalez A., Zhao M., Leavitt J. M. & Lloyd A. M. Regulation of the anthocyanin biosynthetic pathway by the TTG1/bHLH/Myb transcriptional complex in *Arabidopsis* seedlings. Plant J 53, 814–827 (2008). 18036197 10.1111/j.1365-313X.2007.03373.x

[b22] Kobayashi S., Goto-Yamamoto N. & Hirochika H. Retrotransposon-induced mutations in grape skin color. Science 304, 982–982 (2004). 15143274 10.1126/science.1095011

[b23] Ban Y. . Isolation and functional analysis of a MYB transcription factor gene that is a key regulator for the development of red coloration in apple skin. Plant Cell Physiol 48, 958–970 (2007). 17526919 10.1093/pcp/pcm066

[b24] Takos A. M. . Light-induced expression of a *MYB* gene regulates anthocyanin biosynthesis in red apples. Plant Physiol 142, 1216–1232 (2006). 17012405 10.1104/pp.106.088104 PMC1630764

[b25] Zimmermann I. M., Heim M. A., Weisshaar B. & Uhrig J. F. Comprehensive identification of *Arabidopsis thaliana* MYB transcription factors interacting with R/B-like BHLH proteins. Plant J 40, 22–34 (2004). 15361138 10.1111/j.1365-313X.2004.02183.x

[b26] Lin-Wang K. . An R2R3 MYB transcription factor associated with regulation of the anthocyanin biosynthetic pathway in Rosaceae. BMC Plant Biol 10, 50 (2010). 20302676 10.1186/1471-2229-10-50 PMC2923524

[b27] Stracke R., Werber M. & Weisshaar B. The R2R3-MYB gene family in *Arabidopsis thaliana*. Curr Opin Plant Bio 4, 447–456 (2001). 11597504 10.1016/s1369-5266(00)00199-0

[b28] Yildiz M. . Expression and mapping of anthocyanin biosynthesis genes in carrot. Theor Appl Genet 126, 1689–1702 (2013). 23525633 10.1007/s00122-013-2084-y

[b29] Wako T., Kimura S., Chikagawa Y. & Ozeki Y. Characterization of MYB proteins acting as transcriptional regulatory factors for carrot *phenylalanine ammonia-lyase* gene (*DcPAL3*). Plant Biotechnol 27, 131–139 (2010).

[b30] Zhou H. . Molecular genetics of blood-fleshed peach reveals activation of anthocyanin biosynthesis by NAC transcription factors. Plant J 82, 105–121 (2015). 25688923 10.1111/tpj.12792

[b31] Wang Z. G. . The Methylation of the *PcMYB10* Promoter Is Associated with Green-Skinned Sport in Max Red Bartlett Pear. Plant Physiol 162, 885–896 (2013). 23629835 10.1104/pp.113.214700 PMC3668077

[b32] Huang W. J. . A R2R3-MYB transcription factor from *Epimedium sagittatum* regulates the flavonoid biosynthetic pathway. Plos One 8 (2013). 10.1371/journal.pone.0070778 PMC3731294 23936468

[b33] Mano H., Ogasawara F., Sato K., Higo H. & Minobe Y. Isolation of a regulatory gene of anthocyanin biosynthesis in tuberous roots of purple-fleshed sweet potato. Plant Physiol 143, 1252–1268 (2007). 17208956 10.1104/pp.106.094425 PMC1820918

[b34] Xu Z. S., Tan H. W., Wang F., Hou X. L. & Xiong A. S. CarrotDB: a genomic and transcriptomic database for carrot. Database (Oxford) 2014 (bau096), 1–8 (2014). 10.1093/database/bau096 PMC4178371 25267795

[b35] Iorizzo M. . A high-quality carrot genome assembly provides new insights into carotenoid accumulation and asterid genome evolution. Nat Genet 48, 657–666 (2016). 27158781 10.1038/ng.3565

[b36] Clough S. J. & Bent A. F. Floral dip: a simplified method for Agrobacterium-mediated transformation of *Arabidopsis thaliana*. Plant J 16, 735–743 (1998). 10069079 10.1046/j.1365-313x.1998.00343.x

[b37] Li Y.-Y. . MdCOP1 ubiquitin E3 ligases interact with MdMYB1 to regulate light-induced anthocyanin biosynthesis and red fruit coloration in apple. Plant Physiol 160, 1011–1022 (2012). 22855936 10.1104/pp.112.199703 PMC3461526

[b38] Wang G. L. . Regulation of ascorbic acid biosynthesis and recycling during root development in carrot (*Daucus carota* L.). Plant Physiol Bioch 94, 10–18 (2015). 10.1016/j.plaphy.2015.04.014 25956452

[b39] Schmittgen T. D. & Livak K. J. Analyzing real-time PCR data by the comparative C-T method. Nat Protoc 3, 1101–1108 (2008). 18546601 10.1038/nprot.2008.73

