# Supplementary material for: A MYB transcription factor, DcMYB6, is involved in regulating anthocyanin biosynthesis in purple carrot taproots
Source: Sci Rep. 2017 Mar 27;7:45324. doi: 10.1038/srep45324 (PMC5366895; doi:10.1038/srep45324)

**A MYB transcription factor, DcMYB6, is involved in regulating anthocyanin biosynthesis in purple carrot taproots**

Zhi-Sheng Xu, Kai Feng, Feng Que, Feng Wang, Ai-Sheng Xiong*

*State Key Laboratory of Crop Genetics and Germplasm Enhancement, College of Horticulture, Nanjing Agricultural University, Nanjing, 210095, China*

*Please address all correspondence to: A.S. Xiong (xiongaisheng@njau.edu.cn)

-----------------

Dr. Ai-Sheng Xiong

Professor

State Key Laboratory of Crop Genetics and Germplasm Enhancement,

College of Horticulture,

Nanjing Agricultural University,

Nanjing, 210095, China

Tel: 86 25 84396790

Fax: 86 25 84396790

Email: xiongaisheng@njau.edu.cn

***Running title:*** *An anthocyanin biosynthesis related MYB transcription factor from purple carrot*

**Supplementary Information**

**Table S1. GenBank accession numbers of R2R3-MYBs used to build phylogenetic tree.**

| **Name of R2R3-MYBs** | **GenBank accession numbers** |
| --- | --- |
| NtAN2(*Nicotiana tabacum*) | ACO52470 |
| PhAn2 (*Petunia x hybrida*) | AAF66727 |
| LeANT1 (*Lycopersicon esculentum*) | AAQ55181 |
| IbMYB1 (*Ipomoea batatas*) | BAF45114 |
| InMYB2 (*Ipomoea nil*) | BAE94709 |
| VvMYBA1 (*Vitis vinifera*) | BAD18977 |
| VvMYBA2 | BAD18978 |
| *CsRuby* (*Citrus sinensis*) | AFB73913 |
| AmVENOSA (*Antirrhinum majus*) | ABB83828 |
| AmROSEA1 | ABB83826 |
| AmROSEA2 | ABB83827 |
| AtPAP2/MYB90 (*Arabidopsis thaliana*) | AAG42002 |
| AtMYB114 | Q9FNV8 |
| AtPAP1/MYB75 | AAG42001 |
| AtMYB113 | Q9FNV9 |
| GmMYB10 (*Garcinia mangostana*) | ACM62751 |
| MrMYB1 (*Morella rubra*) | ADG21957 |
| MdMYB10a (*Malus × domestica*) | ABB84753 |
| MdMYB1-1 | DQ886414 |
| GhMYB10 (*Gerbera hybrid*) | CAD87010 |
| MtLAP1 (*Medicago truncatula*) | ACN795410 |
| LhMYB6 (*Lilium hybrid*) | BAJ05399 |
| EsMYBA1 (*Epimedium sagittatum*) | AGT39060 |
| ZmC1 (*Zea mays*) | AAA33482 |
| ZmPl | AAA19819 |
| LjTT2a (*Lotus japonicus*) | BAG12893 |
| VvMYBPA2 | ACK56131 |
| FaMYB11 (*Fragaria x ananassa*) | AFL02461 |
| AtTT2 | NP_198405 |
| FaMYB9 | AFL02460 |
| OsMYB3 (*Oryza sativa*) | BAA23339 |
| OsMYB3 (*Oryza sativa*) | BAA23339 |
| DkMYB4 (*Diospyros kaki*) | BAI49721 |
| VvMYBPA1 | CAJ90831 |
| AtMYB32 | EFH43356 |
| AtMYB4 | NP_195574 |
| AtMYB7 | NP_179263 |
| AtMYB12 | ABB03913 |
| SlMYB12 | ACB46530 |
| VvMYBF1 | ACV81697 |
| VvMYB5a | AAS68190 |
| VvMYB5b | AAX51291 |

**Table S2. List of primers used for qRT-PCR analyses of transgenic *Arabidopsis thaliana***

| **Gene name** | **Gene identifier** | **Forward primer (5’-3’)** | **Reverse primer (5’-3’)** |
| --- | --- | --- | --- |
| *AtCHS* | AT5G13930.1 | GCATCTTGGCTATTGGCACTG | CGTTTCCGAATTGTCGACTTGT |
| *AtCHI* | AT3G55120.1 | CTCCTCCAATCCATTATTCCTCG | TTTCCCTTCCACTTGACAGATAGAG |
| *AtF3H* | AT3G51240.1 | GTGTTTAGCGACGAAATCCCG | ACGAGCGAGACGAGTCATATCC |
| *AtF3'H* | AT5G07990.1 | TCGTGGTCGCCGCTTCTAA | CCATCGGTGTCCGTAAGGTG |
| *AtDFR* | AT5G42800.1 | CAAACGCCAAGACGCTACTCA | CATTCACTGTCGGCTTTATCACTTC |
| *AtLDOX* | AT4G22880.1 | ACGGTCCTCAAGTTCCCACAA | CAGCTCCTCAATACAATTCTCACG |
| *AtUGT78D2* | AT5G17050.1 | ACCGCACAATCCAACTCTTCG | TCCTGTGGTCTCCCGCTAAA |
| *AtACT2* | AT3G18780.1 | AACCACTATGTTCTCAGGTATCGCT | TGGACCTGCCTCATCATACTCG |

**Figure S1. Alignment analysis of ORF and genomic DNA sequences and genomic structure of *DcMYB6* from ‘Deep purple’ carrot.**

(A) Alignment analysis of cDNA and gDNA sequences of *DcMYB6*. (B) Genomic structure of *DcMYB6* consisted of three exons and two introns, the exons and introns are shown as yellow blocks and black lines, respectively.


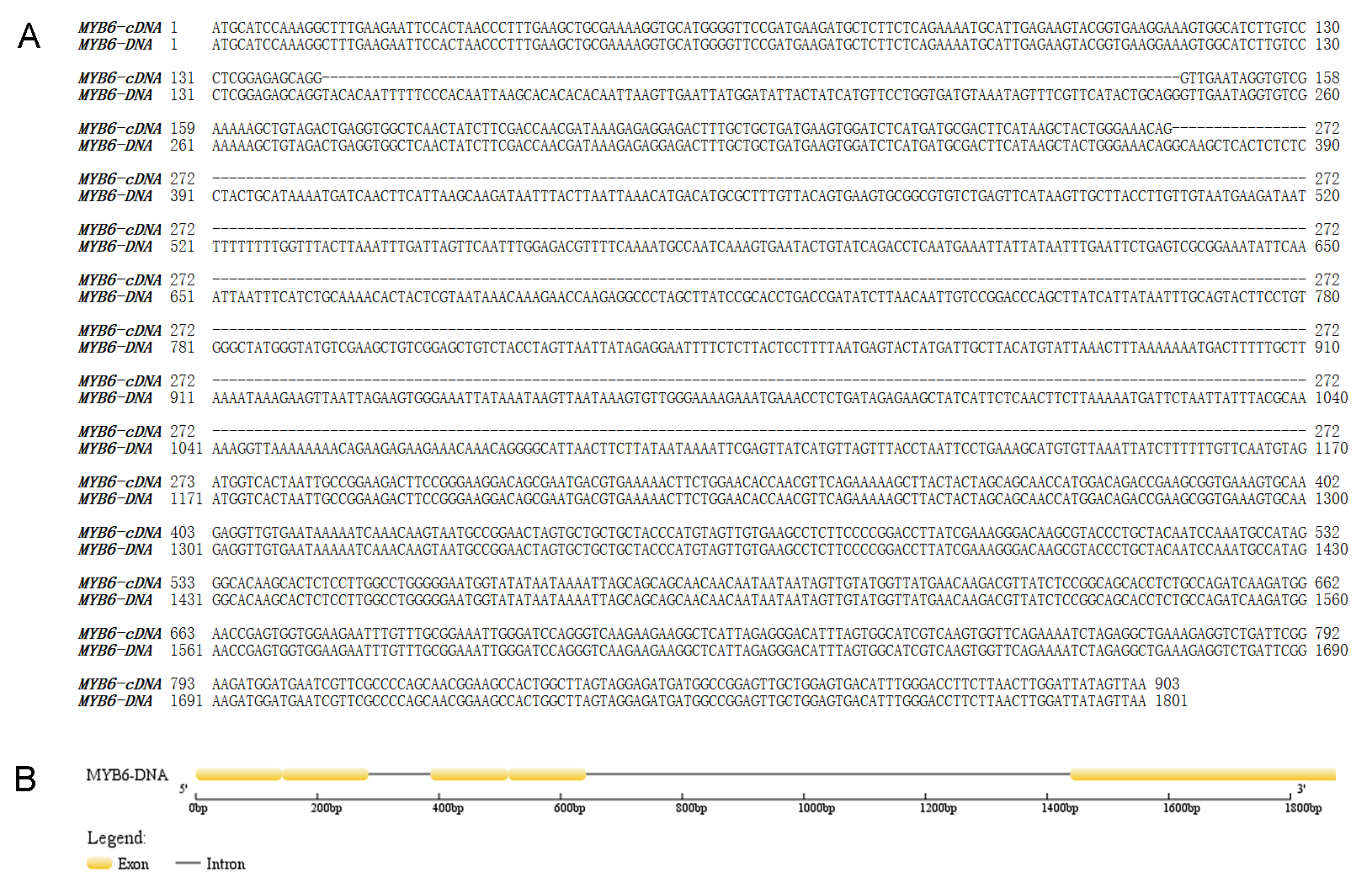

Supplement: Supplementary Information [file srep45324-s1.doc]
